# Promoting Effects of Different Organic Acids on the Formation of Transglutaminase-Induced Cross-Linked Soybean Protein Isolate Hydrogels

**DOI:** 10.3390/foods14111965

**Published:** 2025-05-31

**Authors:** Xiangquan Zeng, Linlin Peng, Sirong Liu, Haoluan Wang, He Li, Yu Xi, Jian Li

**Affiliations:** 1 Key Laboratory of Geriatric Nutrition and Health, Ministry of Education, Beijing Technology and Business University, Beijing 100048, China; 20210803@btbu.edu.cn (X.Z.); 19834545170@163.com (L.P.); 15223376402@163.com (S.L.); 15511316985@163.com (H.W.); lihe@btbu.edu.cn (H.L.); fenglin5377@126.com (Y.X.); 2 Key Laboratory of Green and Low-Carbon Processing Technology for Plant-Based Food of China National Light Industry Council, School of Food and Health, Beijing Technology and Business University, Beijing 100048, China; 3 Beijing Engineering and Technology Research Center of Food Additives, School of Food and Health, Beijing Technology and Business University, Beijing 100048, China

**Keywords:** soybean protein isolate, transglutaminase, organic acids, hydrogels, gel properties, interactions

## Abstract

Microbial transglutaminase (mTG) is most frequently utilized in order to increase the gelling properties of soybean protein isolate (SPI), but there are still some limitations of mTG-based hydrogel fabrication technology. Therefore, we aimed to develop a dual modification technique based on enzyme plus organic acid treatment to fabricate SPI hydrogels with high gel strength and stability. Our results showed that mTG plus glucose-δ-lactone (GDL), lactobionic acid (LBA) or maltobionic acid (MBA) treatment could significantly improve the gel strength, textural properties, and water-holding capacity of SPI hydrogels. Also, the addition of these organic acids remarkably reduced the surface hydrophobicity (H_0_) and intrinsic fluorescence as well as increased the storage modulus (G′), loss modulus (G″) values, average particle size, and the absolute value of zeta potential of samples. GDL, LBA, or MBA greatly increased the β-sheet level and decreased the α-helix level in hydrogels, as well as dissociated 11S subunits of SPI into 7S subunits. Notably, covalent interactions, hydrogen bonding, and hydrophobic interactions of three organic acids with SPI, as well as the effects of organic acids on the interactions among the intramolecular and intermolecular forces of SPI molecules, contributed to their promoting effects on the formation of hydrogels. The LF-NMR and SEM analyses confirmed the effects of GDL, LBA, and MBA on converting the free water into immobilized and bound water as well as forming a dense stacked aggregate structure. Therefore, GDL, LBA, and MBA are promising agents to be combined with mTG in the fabrication of SPI hydrogels with high gel strength and stability.

## 1. Introduction

As a highly nutritious plant protein, soybean protein isolate (SPI) is widely applied in the food industry due to its good gel properties [1]. Nowadays, a variety of soybean protein gel-based food products can be found in the market, such as plant-based meat analogs, sausages, yogurt, tofu, and puddings [2]. During the formation of SPI hydrogels, heat treatment of SPI is a key step. The protein aggregates formed in the heating process are essential to the nature of SPI hydrogels [3]. Nevertheless, the stability of hydrogels fabricated solely based on the gel properties of SPI is relatively low during food processing and storage, which is unable to satisfy the producers and consumers all the time [4]. Therefore, different treatments are utilized to enhance the gel properties of SPI and improve the gel networks of these hydrogels, including treatment with enzymes, acids, salts, etc. [5]. Enzyme treatment is one of the most common methods among these treatments, in which transglutaminase (TG), laccase, glucose oxidase, and some endogenous enzymes can be made use of [6]. Notably, TG is the most frequently utilized during the fabrication of SPI hydrogels since it can promote the gelation of SPI by cross-linking protein molecules [7]. Transglutaminases are categorized as animal-derived (e.g., liver tissues) or microbial-derived, with microbial TGase (mTG) being industrially preferred for its broader pH/temperature adaptability, wider substrate specificity, and more cost-effective production [8]. To be specific, mTG is able to catalyze the amido-group transfer reaction between the γ-amide group of glutamine residues in proteins and the ε-amino group of lysine to form the heterotype peptide bond of ε-(γ-glutamine)-lysine, thus changing the gelling properties of SPI. This cross-linking effect has been similarly demonstrated in other plant protein systems—Nivala et al. [9] reported that heat and transglutaminase treatment of faba bean protein yielded gels with enhanced mechanical strength, characterized by elevated storage modulus and strain hardening response, indicating mechanically strong gels. In the study of Sun and Arntfield [10], it was found that the strength of pea protein or soy protein hydrogels increased by approximately eight-fold and one-fold after treatment with TG. Although mTG-based SPI hydrogel fabrication technology has been popular among producers in recent years, there are still some issues that need to be resolved, like poor heat resistance of mTG during application, the unsatisfactory gel strength, and low gel stability of SPI hydrogels prepared by mTG. Therefore, dual modification techniques are introduced to increase the efficiency of enzyme treatment to produce protein hydrogels with high gel strength and stability [11].

**Figure 1:**
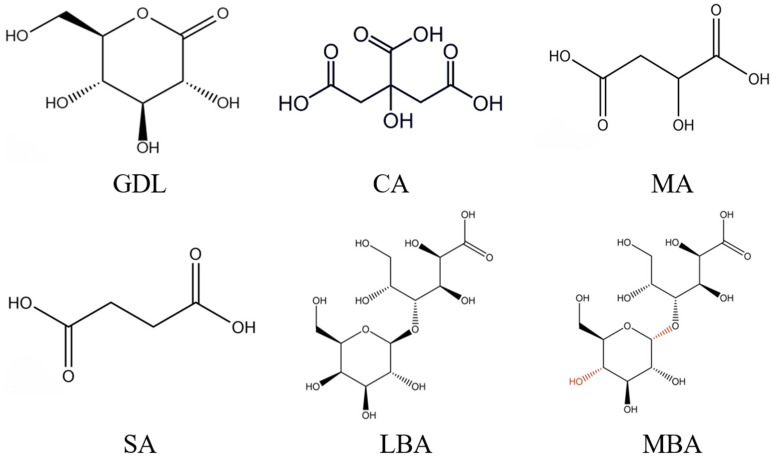
Chemical structures of GDL, CA, MA, SA, LBA, and MBA.

Acid treatment was another common technology to prepare SPI hydrogels. It is worth mentioning that acid-induced gels are driven by interactions formed by hydrophobic amino acids in SPI after the formation of hydrated hydrogen ions that neutralize repulsive charges and reduce spatial repulsion [12,13]. Nicole et al. [14] evaluated the gelation behavior and properties of SPI treated by calcium sulfate and GDL; they found that the SPI hydrogels treated by GDL showed a shorter gelation time and higher energy storage moduli than those treated by calcium sulfate. Due to the high convenience and efficiency of acid treatment, nowadays, researchers have begun to combine this technology with enzyme treatment to further improve the gelling properties of SPI hydrogels. For instance, Bersanetti et al. [15] fabricated a novel hydrogel using polyvinyl alcohol (PVA), ferulic acid, and laccase and observed that ferulic acid was able to form a network with PVA and significantly increase the viscosity of hydrogels treated by laccase. In previous studies, various organic acids have been applied in the preparation of protein gels, such as glucose-δ-lactone (GDL), citric acid (CA), malic acid (MA), succinic acid (SA), lactobionic acid (LBA), and maltobionic acid (MBA) (Figure 1) [16,17]. GDL is an acidic coagulant that can promote gel formation by gradually lowering the pH of protein to the isoelectric point through the continuous release of protons in the protein solution [13]. CA and MA originate from fruits and vegetables, which are mild water-soluble acidifying agents and have been widely applied in the food industry [18]. Finally, SA can be used as a flavor enhancer, softener, or catalyst in beverages, baked food, or the preparation of food seasonings, respectively [19]. As an aldehydic acid with a sweet taste, LBA is usually applied in food products as an antioxidant, gelling agent, or stabilizer [20,21]. MBA is a stereoisomer of LBA with physicochemical properties similar to those of LBA [22]. Nevertheless, few researchers attempted to combine these organic acids with mTG treatment to enhance the gelling properties of SPI hydrogels and further explore the promoting effects.

Consequently, our investigation aimed to develop a novel dual modification technique based on enzyme plus acid treatment to fabricate SPI hydrogels. Specifically, the promoting effects of different organic acids (GDL, CA, MA, SA, LBA, and MBA) on the formation of mTG-induced cross-linked SPI hydrogels were compared, while the involved mechanisms were further explored. Our findings might significantly improve the gelling properties of mTG-induced cross-linked SP hydrogels and broaden the applications of SPI hydrogels in food production and processing.

## 2. Materials and Methods

### 2.1. Chemicals

SPI was provided by Yuwang Industrial Co., Ltd. (Yucheng, China), while mTG (100 U/g) was purchased from Dongsheng Biotechnology Co., Ltd. (Zhongshan, China). GDL was obtained from Shanghai Yuanye Biotechnology Co., Ltd. (Shanghai, China), while CA, MA, SA, LBA, and MBA were purchased from Shanghai McLean Biochemistry and Technology Co., Ltd. (Shanghai, China). Other reagents were analytical grade, and deionized water was used throughout the experiment.

### 2.2. Preparation of SPI Hydrogels

SPI-hydrogels were prepared based on the methods of Zeng et al. [23] and Liang et al. [24], with some modifications. Briefly, 12.0 g of SPI powder was mixed with 100 mL of distilled water and then stirred at 500 rpm at room temperature for 120 min. Subsequently, mTG (40 U/g) was added to the mixture, and then different amounts of GDL, CA, MA, SA, LBA, or MBA were added to obtain a final acid concentration of 0.0, 0.2, 0.4, 0.6, 0.8, or 1.0% (*w*/*v*), respectively. The mixtures were kept in a water bath of 40 °C for 60 min, after which they were heated in a water bath of 95 °C for 30 min. Ultimately, the samples were cooled in an ice water bath for 10 min and stored at 4 °C overnight.

### 2.3. Gel Strength Analysis

The gel strength of different samples was determined using a texture analyzer (FTC Corporation, Rockville, Maryland, USA, TMS-Touch) according to the methods of Li et al. [25], with minor modifications. A cylindrical plastic probe with a diameter of 12.7 mm was used to squeeze the gel. The parameters were set as follows: test speed at 3.0 mm/s, starting force at 0.1 N, and puncture distance of 10 mm.

### 2.4. Textural Analysis

In terms of texture analysis, the hardness, elasticity, cohesion, and chewability of the SPI hydrogels were determined using the texture analyzer (FTC Corporation, USA, TMS-Touch) with a 38.1 mm cylindrical probe. The testing speed, compression ratio, trigger force, and rise distance for texture analysis were 3.0 mm/s, 50%, 0.1 N, and 20 mm, respectively.

### 2.5. Water Holding Capacity (WHC) Analysis

The WHC of different samples was determined as described by Jian et al. [26], with minor modifications. Specifically, 5 g of hydrogel was weighed and placed in a dry centrifuge tube, then it was centrifuged at 1000× *g* for 15 min at 4 °C using a centrifuge (3–30 K high-speed tabletop centrifuge, SIGMA, Osterode am Harz, Lower Saxony, Germany). After centrifugation, excess water was removed and the mass of hydrogel was determined. The WHC (%) of the sample was calculated as follows:

(1) $$\mathrm{WHC}(\%)=\frac{m_{2}-m_{0}}{m_{1}-m_{0}}\times 100$$

where m_0_ was the weight of the empty centrifuge tube (g), while m_1_ or m_2_ was the weight of the sample before or after centrifugation (g), respectively.

### 2.6. Rheological Analysis

The rheological properties of different SPI hydrogels were characterized based on the method of Liu et al. [27], with minor modifications. The measurements were conducted using a stress-controlled rheometer (DHR-1, TA Instruments, New Castle, DE, USA) with Peltier temperature control system at 37 °C. Central portions of the hydrogels were carefully excised and trimmed to 30 mm × 30 mm × 10 mm dimensions before being positioned at the center of the lower plate. The upper plate was then carefully adjusted to maintain 1 mm gap distance, with excess material being trimmed away using a scraper. A linear viscoelastic region with a strain value of 1% was selected, while the scanning speed ranged from 0.1 to 60 rad/s. In the rheological analysis, the energy storage modulus (G′) and loss modulus (G″) of samples were determined.

### 2.7. Surface Hydrophobicity (H_0_) Analysis

The H_0_ value of samples was investigated according to the method of Fu et al. [28] using 8-aniline-1-naphthalenesulfonic acid (ANS) as a fluorescent probe. To be specific, protein solution at different concentrations (0.1–1 mg/mL) was prepared from lyophilized powder with phosphoric acid buffer (0.01 mol /L, pH 7.0), then 4 mL protein solution was mixed with 40 μL ANS (8 mmol/L) to react for 3 min in the dark. The fluorescence intensity of ANS was detected by a microplate reader (TECAN, Männedorf, Zurich, Switzerland) at an excitation wavelength of 390 nm and an emission wavelength of 470 nm. The linear relationship between fluorescence intensity and protein concentration was plotted, while the initial slope is surface hydrophobicity.

### 2.8. Zeta Potential and Particle Size Analysis

The zeta potential and particle size of samples were determined using a Zetasizer Nano ZS 90 (Malvern Instrument Ltd., Worcestershire, UK) particle size analyzer. According to Zhao et al. [29], the lyophilized powder of hydrogels was dissolved in distilled water to obtain the hydrogel solution at a concentration of 1 mg/mL. Subsequently, it was filtered through a 0.45 μm filter membrane and used for zeta potential and particle size analysis.

### 2.9. Fluorescence Spectroscopy Analysis

An FS5 fluorescence spectrophotometer (Applied Photophysics Ltd., Edinburgh, UK) was utilized to conduct the fluorescence spectroscopy analysis [30]. Different hydrogel solutions (1.0 mg/mL) were prepared using phosphate buffer (0.01 mol/L, pH 7.0). The excitation wavelength of the fluorescence spectra was set at 280 nm, while the scanning range of the emission wavelength or the width of both the excitation and emission slits was 320–450 nm or 5 nm, respectively.

### 2.10. Fourier Transform Infrared (FT-IR) Spectroscopy Analysis

To investigate the secondary structure of SPI hydrogels, an FT-IR spectrometer equipped with an attenuated total reflection Fourier transform (ATR-FT, Thermo Fisher Scientific Inc., Rochester, NY, USA) device was utilized. The scanning range was between 400 and 4000 cm^−1^ while the scanning was conducted with 32 signal scanning accumulations at a resolution of 4 cm^−1^. Advanced ATR correction (diamond) was performed using Omnic software, while peak splitting was fitted to the amide I band in the range of 1700–1600 cm^−1^ of the spectrum of samples using Peakfit 4.12 software to identify the correspondence between the position of each subpeak and the secondary structures.

### 2.11. Sodium Dodecyl Sulfate-Polyacrylamide Gel Electrophoresis (SDS-PAGE) Analysis

Based on the method of Chen, Zhang, Zhang and Wang [31], 5 mg of sample powder was dissolved in 0.5 mL of loading buffer [10% (*w*/*w*) of SDS, 1% (*w*/*w*) of bromophenol blue, glycerin, β-mercaptoethanol, and 1 M Tris-HCl] and then vortexed completely. After 1 h, the sample solution was heated in a boiling water bath for 10 min. and then cooled at room temperature. Subsequently, it was centrifuged at 10,000 rpm for 5 min, and the supernatant was collected. A total of 12.5% of separator gel and 4.5% of concentrate gel were used for the SDS-PAGE analysis, while the molecular weights of the marker ranged from 11–180 kDa. Ultimately, protein electrophoresis patterns were captured using a gel imaging system.

### 2.12. Protein Interaction Forces Analysis

To explore the roles of intramolecular and intermolecular forces in the formation of SPI hydrogels, the changes in protein solubility were characterized after extracting with eight different buffer systems [32]. Eight buffer systems were ① phosphate buffer (P) (0.035 mol/L, pH 7.6), ② P + Urea (8 mol/L), ③ P +2-mercaptoethanol (2-ME) (0.1 mol/L), ④ P + SDS (1.5%, m/V), ⑤ P + Urea + 2-ME, ⑥ P + Urea + SDS, ⑦ P + SDS + 2-ME, and ⑧ P + Urea + SDS + 2-ME, respectively. The contents of various chemical bonds were calculated by measuring the solubility of samples in different solvents according to the following equations: natural state protein (①), hydrogen bond (② − ①), disulfide bond (③ − ①), hydrophobic interaction (④ − ①), hydrogen bond and disulfide bond interaction (⑤ + ① − ② − ③), hydrogen bond and hydrophobic interaction interactions (⑥ + ① − ② − ④), hydrogen bond, hydrophobic interaction, and disulfide bond interaction (⑧ + ② + ③ + ④ − ① − ⑤ − ⑥ − ⑦). Total protein content of samples was quantified by Kjeldahl method, while soluble protein content was determined by Lowery method after extraction.

### 2.13. Low-Field Nuclear Magnetic Resonance (LF-NMR) and Magnetic Resonance Imaging (MRI) Analysis

An LF-NMR analyzer (MesoMR12-060H-HTHP, Niumag Corporation, Suzhou, China) was used to measure the moisture distribution of different SPI hydrogels in our study. In particular, 4 g of sample was weighed into a 25 mm NMR glass tube, and the CPMG (Carr–Purcell–Meiboom–Gill) pulse sequence was used for the test. The acquisition parameters of T_2_ were as follows: spectral width (100 kHz), number of samples (59,990), 180° pulse width (14 μs), number of echoes (2000), echo time (0.300 ms), waiting time (2000 ms), and cumulative number (4 times). The data were acquired by NMR analysis software, while the SIRT method was applied for inversion processing with the relaxation time ranging from 0.01 ms to 10,000 ms.

Regarding MRI analysis, 4 g of samples were placed in a 50 mm NMR tube and then transferred to a low-field NMR analyzer. The proton density imaging of different hydrogels was obtained using a multi-layer spin echo sequence (Multi-SliceEcho, MSE,) Siemens Healthineers, Erlangen, Germany. Moreover, the scanning program was set up with a repeat wait time (TR) of 400 ms, an echo time (TE) of 20 ms, and an averaging number of 4 times. Ultimately, the images of samples were saved in PNG format, while the pseudo-color processing was uniformly performed using Newmark MRI image processing software (version 2.5).

### 2.14. Scanning Electron Microscopy (SEM) Analysis

The microstructures of different SPI hydrogels were observed using an SU8020 scanning electron microscope (SEM, Hitachi High-Technologies Corporation, Tokyo, Japan). Briefly, the freeze-dried samples were fixed on a sample stage with conductive adhesive, sprayed with gold under vacuum using a spray coating machine, and the microstructures were observed with a scanning electron microscope (Zeiss SUPRA 55, Carl Zeiss AG, Oberkochen, Germany) at 20 kv.

### 2.15. Statistical Analysis

All experiments and analyses were repeated three times, and results were expressed as mean ± standard deviation (SD) using SPSS 21.0 (USA) for Windows. The statistical significance of data was determined using one-way analysis of variance (ANOVA) followed by the comparison of Duncan’s Multiple Range Test (DMRT); *p* values < 0.05 were regarded as significant.

## 3. Results and Discussion

### 3.1. Effects of Different Organic Acids on the Gel Strength of mTG-Induced Cross-Linked SPI Hydrogels

**Figure 2:**
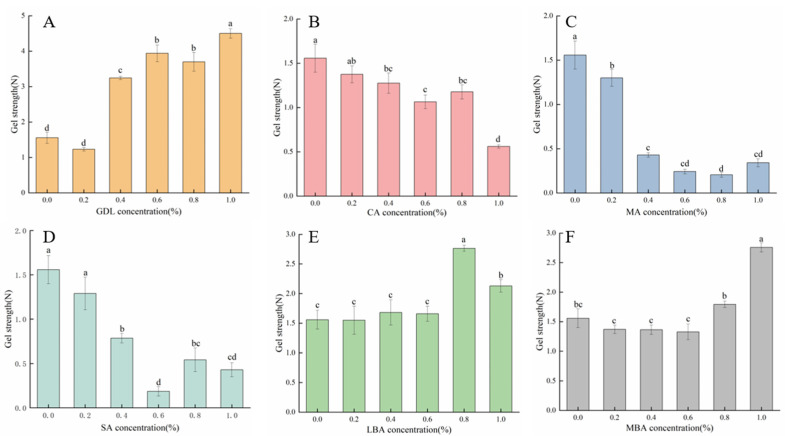
Effect of GDL (**A**), CA (**B**), MA (**C**), SA (**D**), LBA (**E**), and MBA (**F**) on the gel strength of mTG-induced cross-linked SPI hydrogels. (Note: Data are presented as mean ± SD, *n* = 3, 95% CI). Lowercase letters represent significant differences (*p* < 0.05).

Gel strength is an important index reflecting the functional properties of hydrogels [33]. Figure 2 shows the effects of different organic acids on the gel strength of mTG-induced cross-linked SPI hydrogels. It was observed that the gel strength of mTG-induced cross-linked hydrogels significantly increased with the rising concentrations of GDL, LBA, and MBA (0–1.0% *w*/*v*), while the gel strength of GDL plus mTG treatment was relatively higher than that of LBA plus mTG or MBA plus mTG treatment. Notably, the gel strength of SPI hydrogels reached 4.50 N and 2.16 N at GDL and MBA concentrations of 1.0% (*w*/*v*), respectively, whilst the gel strength of SPI hydrogels at an additive amount of LBA of 0.8% (*w*/*v*) was 176.9% that of the control. According to previous studies, organic acid treatments could not only weaken the electrostatic repulsion between protein molecules or aggregates but also reduce the pH of the gel system to approach the isoelectric point of the proteins, which further enhanced the strength of protein hydrogels [34]. By contrast, the addition of CA, MA, and SA (0–1.0% *w*/*v*) gradually reduced the gel strength of mTG-induced cross-linked SPI hydrogels due to their adverse effects on gel network structures. When the concentration of CA, MA, or SA was 1.0%, the gel strength of hydrogels was only 36.0%, 22.0%, or 27.6% that of the control, respectively. According to the results of previous studies, the gel strength of hydrogels might be related to the rate of acidification. The hardness of hydrogels remarkably decreased with the increase in acidification rate due to the decrease in time required for the formation and alignment of bonds between the proteins [35]. It seemed that the increase in the rate of acidification of mTG-induced cross-linked SPI hydrogels after treatment with CA, MA, or SA resulted in a decrease in gel strength.

### 3.2. Effects of Different Organic Acids on the Textural Properties of mTG-Induced Cross-Linked SPI Hydrogels

The effects of different organic acids on the textural properties of mTG-induced cross-linked SPI hydrogels were investigated in our study as well. As shown in Table 1, the addition of GDL, LBA, or MBA (0–1.0% *w*/*v*) significantly increased the hardness, cohesion, springiness, and gumminess of mTG-induced cross-linked SPI hydrogels, while the values of these parameters of samples continuously decreased after treating with CA, MA, or SA. The findings of textural analysis were in line with those of gel strength analysis. It was worth mentioning that the values of textural parameters for mTG plus GDL treatments were the highest among all treatments. When the concentration of GDL was 1.0% (*w*/*v*), the hardness, cohesion, springiness, or gumminess of hydrogels was 275.5%, 147.1%, 326.4%, or 521.2% higher compared with that of the control, respectively. Based on the study of Yang et al. [36], the acidification rate of GDL was lower than that of CA, which contributed to the higher hardness and elasticity of GDL-induced SPI hydrogels. Hence, it could be hypothesized that the better gel strength and textural properties of mTG-induced cross-linked SPI hydrogels treated with GDL, LBA, or MBA were probably associated with the relatively lower acidification rate of these organic acids.

### 3.3. Effects of Different Organic Acids on the WHC of mTG-Induced Cross-Linked SPI Hydrogels

**Figure 3:**
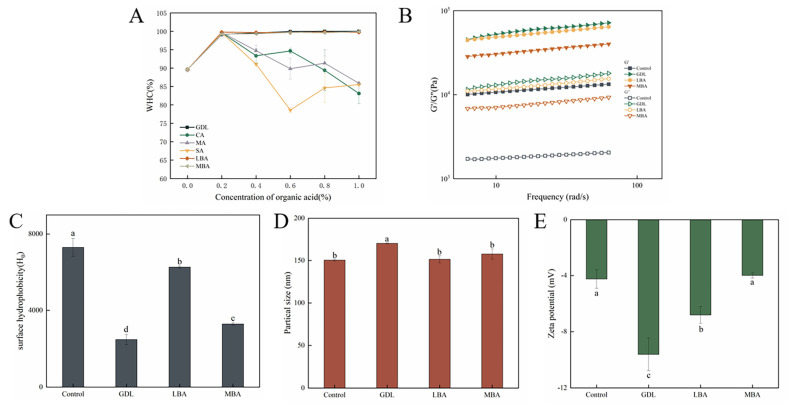
Effect of different organic acids on the WHC (**A**), rheological properties (**B**), H_0_ (**C**), average particle size (**D**), and zeta potential (**E**) of mTG-induced cross-linked SPI hydrogels. (**B**–**E**: 1.0% GDL, 0.8% LBA, and 1.0% MBA *w*/*v*). Lowercase letters represent significant differences (*p* < 0.05). (Note: Data are presented as mean ± SD, *n* = 3, 95% CI.)

WHC is an index for evaluating the processing characteristics of hydrogels, which responds to the ability of hydrogels to bind water as well as the homogeneity and denseness of the gel network structure. The effects of different organic acids on the WHC of mTG-induced cross-linked SPI hydrogels are presented in Figure 3A. It was shown that the addition of GDL, LBA, or MBA remarkably increased the WHC of hydrogels, and the WHC of samples was almost 100% when the concentration of GDL, LBA, or MBA was higher than 0.2% (*w*/*v*). Although treating with 0.2% (*w*/*v*) of CA, MA, or SA slightly increased the WHC of SPI hydrogels as well, the WHC sharply decreased when the concentration of these organic acids increased from 0.2% (*w*/*v*) to 1.0% (*w*/*v*). It seemed that CA, MA, or SA treatment might loosen the structure of SPI hydrogels to reduce their WHC, thus greatly increasing the free water content. Yang et al. [36] found that the WHC of hydrogels was correlated with the acidification rate of organic acids, while the acid with a higher acidification rate could induce the formation of looser hydrogels with lower WHC, likely due to accelerated protein aggregation. In the research of Wang et al. [37], it was demonstrated that the structure of hydrogels played a crucial role in the retention of water, while a strong and homogeneous gel structure was able to lead to a tighter encapsulation of water within the gel matrix. In short, the increase in WHC of mTG-induced cross-linked hydrogels treated with GDL, LBA, and MBA might be attributed to their ability to improve the gel strength and textural properties of hydrogels, which resulted in a more robust structure and tighter bonding with water molecules.

Lowercase letters represent significant differences (*p* < 0.05).

On the basis of gel strength, textural and WHC analysis, GDL, LBA, and MBA significantly improve the gel strength, textural properties, and WHC of mTG-induced cross-linked SPI hydrogels, while the gel network structures were greatly destroyed after treating with CA, MA, and SA. Notably, treatment with 1.0% (*w*/*v*) of GDL, 0.8% (*w*/*v*) of LBA, or 1.0% (*w*/*v*) of MBA induced the formation of hydrogels with high gel strength and WHC, as well as satisfactory textural properties. Therefore, in the subsequent studies, these three organic acids at their optimal concentrations were selected as the experimental parameters of the dual modification technique to fabricate innovative complex gels and further explore the promoting effects on the formation of mTG-induced cross-linked SPI hydrogels.

### 3.4. Rheological Properties, H_0_, Particle Size, and Zeta Potential of mTG-Induced Cross-Linked SPI Hydrogels Treated with GDL, LBA, or MBA

The rheological properties of hydrogels are essential for their processing and storage [38]. Figure 3B shows the rheological properties of mTG-induced cross-linked SPI hydrogels treated with GDL, LBA, or MBA. It could be seen that the G′ values of all hydrogels were greater than their G″ values, indicating their solid-like natures and the predominance of the elasticity of these samples. Meanwhile, the G′ values of all samples showed a slight frequency dependence, which might be due to the regulating effects of hydrophobic and hydrogen bonding interactions on the gel structures [39]. In the study of Liu et al. [40], The presence of GDL significantly increases the G′ of whey protein isolate and improves its gelation properties. In this study, the addition of GDL, LBA, and MBA induced a significant increase in both G′ and G″ values of mTG-induced cross-linked SPI hydrogels; the GDL-treated samples exhibited the highest moduli values, followed by LBA, with MBA showing the lowest, demonstrating that these organic acids effectively strengthened the gel network structure. The results were in accordance with those of gel strength and textural analysis, suggesting that the addition of three organic acids promoted the formation of denser gel structures of samples [13].

The degree of unfolding of protein structures and the number of exposed surface hydrophobic groups can be reflected by the H_0_ value of hydrogels [41]. The effects of GDL, LBA, and MBA on H_0_ of mTG-induced cross-linked SPI hydrogels were shown in Figure 3C. GDL, LBA, or MBA treatment remarkably reduced the H_0_ of hydrogels, and the H_0_ value of mTG plus GDL treatment was 33.9% that of the control. According to the investigation of Mozafarpour et al. [42], the decrease in H_0_ value was brought about by the formation of protein aggregates through the enhancement of protein–protein intermolecular hydrophobic interactions, which covered the hydrophobic sites on the surface of the proteins and further prevented their binding interactions with the ANS fluorescent probe, thus leading to a decrease in fluorescence intensity. It was inferred that the addition of GDL, LBA, and MBA could promote the aggregation of protein molecules to form more stable SPI hydrogels. Notably, while all three additives effectively induced protein aggregation, the synergistic effect of mTG with GDL demonstrated superior masking efficiency of hydrophobic sites compared with LBA or MBA.

The degradation and aggregation of protein in hydrogels can be visualized by particle size analysis. It has been shown in Figure 3D that the addition of GDL, LBA, and MBA was able to increase the average particle size of mTG-induced cross-linked SPI hydrogel solution, with the GDL-treated group exhibiting a particularly significant increase in particle size. The average particle size of the mTG plus GDL treatment was 1.13-fold that of the control. On the basis of previous studies, the denaturation and aggregation of protein molecules could lead to an increase in average particle size [43,44]. Thus, the increase in particle size of SPI hydrogels treated with the three organic acids was attributable to their promotion of protein molecular aggregation. Zeta potential is the potential at the shear plane between the suspended solid particles and the liquid phase, the absolute value of which suggests the stability of the suspension solution [38]. As shown in Figure 3E, the addition of GDL and LBA significantly increased the absolute value of the zeta potential of mTG-induced cross-linked SPI hydrogel solution, while there was no difference in the absolute value of the zeta potential between mTG plus MBA treatment and control. The absolute value of the zeta potential of hydrogels treated with GDL or LBA was 226.7% or 160.6% that of the control, respectively. It has been revealed that the positive charge on the surface of whey proteins could be remarkably reduced by acid treatment [45]. Therefore, treating with GDL or LBA might increase the absolute value of the negative zeta potential of SPI hydrogels by reducing the positive charge on their surface, which further enhances the stability of complex hydrogels.

### 3.5. Fluorescence Spectroscopy, FT-IR, and SDS-PAGE Analysis of mTG-Induced Cross-Linked SPI Hydrogels Treated with GDL, LBA, or MBA

**Figure 4:**
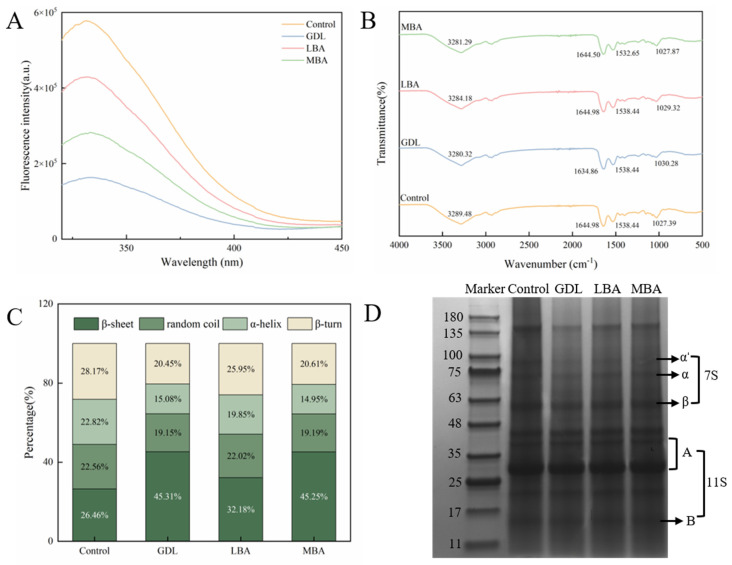
Effects of GDL, LBA, and MBA (1.0%, 0.8%, 1.0% *w*/*v*) on endogenous fluorescence intensity (**A**), FT-IR (**B**), secondary structure content (**C**), and SDS-PAGE images (**D**) of mTG-induced cross-linked SPI hydrogels. (Note: *n* = 3, 95% CI).

The effects of the addition of GDL, LBA, and MBA on the intrinsic fluorescence of mTG-induced cross-linked SPI hydrogels are shown in Figure 4A. It was well accepted that the intrinsic fluorescence of protein hydrogels is usually caused by aromatic amino acids, including tryptophan, tyrosine, and phenylalanine [46]. The change in the intrinsic fluorescence can reflect the changes in conformation and polarity around protein molecules [47]. After treatment with three organic acids, the intrinsic fluorescence intensity of SPI hydrogels significantly decreased. The fluorescence intensity of the mTG plus GDL-treated group was the lowest among all groups, which was only 28.3% that of the control. Wu et al. [48] put forward that heat treatment was able to cause the expansion of the tertiary structure of proteins as well as the exposure of the reactive groups, further promoting the aggregation behavior of protein molecules. Based on our data of H_0_ analysis, the addition of GDL, LBA, and MBA promoted the aggregation of SPI molecules as well, which buried their tryptophan residues and enabled the spatial structure to become more compact and stable, ultimately reducing the intrinsic fluorescence of hydrogels [49,50].

To investigate the interactions of functional groups of GDL, LBA, and MBA with SPI, the FT-IR spectra of different samples were characterized in our research. As shown in Figure 4B, the absorption peaks in the 3200–3500 cm^−1^, 1600–1700 cm^−1^, and 1500–1600 cm^−1^ regions of all hydrogels were attributed to the stretching vibrations of -OH and -NH groups in the amide A band, the stretching vibrations of -C=O in the amide I band, and the bending vibrations of the N-H planar plane and the stretching vibrations of C-N in the amide II band, respectively [51,52]. Compared with the spectrum of control, a blue shift of the peaks related to the amide A band in the spectra of hydrogels treated with GDL, LBA, or MBA was observed. The results indicated the hydrogen-bonding or covalent interactions (formation of ester and amide bonds) of -OH or -NH in the amide A band of SPI with -OH, -C=O, and -C-O-C- groups or -COOH groups of GDL, LBA, or MBA. Moreover, the characteristic peak of the amide I band shifted from 1644.98 cm^−1^ to 1634.86 cm^−1^ in the spectrum of mTG plus GDL treatment, while the peak of the amide II band in the spectrum of mTG plus MBA treatment shifted from 1528.44 cm^−1^ to 1532.65 cm^−1^, suggesting the hydrogen-bonding or covalent interactions of -C=O, N-H, and C-N groups in proteins with -OH, -C=O, -C-O-C-, or -COOH groups of GDL or MBA [53]. As a consequence, the hydrogen-bonding and covalent interactions of three organic acids with SPI might play crucial roles in their promoting effects on the formation of hydrogels with high gel strength.

Further, the effects of three organic acids on the relative contents of protein secondary structures in mTG-induced cross-linked hydrogels were investigated. It has been shown in Figure 4C that the addition of GDL, LBA, and MBA significantly increases the β-sheet level as well as decreases the level of α-helix, β-sheet, and random coil in SPI hydrogels. The β-sheet content in the mTG plus GDL, mTG plus LBA, or mTG plus MBA treatment was 171.1%, 121.6%, or 171.0% that of the control, respectively. According to previous studies [54,55], the β-sheet structure was the basis of protein aggregation and gel formation, while higher β-sheet content and lower α-helix content were closely associated with the enhanced textural properties of proteins and gel network structures. Therefore, the incorporation of three organic acids could promote better gel structure formation in mTG-induced cross-linked SPI hydrogels by affecting the conformation of SPI and unfolding its structure to increase the β-sheet level and decrease the level of α-helix.

The effects of GDL, LBA, and MBA on the SPI subunits of hydrogels are shown in Figure 4D. As the main components of SPI. β-conglycinin (7 S) is a trimeric glycoprotein composed of α′, α, and β subunits, with molecular weights of approximately 80 kDa, 70 kDa, and 50 kDa, respectively, while glycinin (11 S) is a hexameric glycoprotein composed of acidic subunit A (35–41 kDa) and basic subunit B (20 kDa) [56]. There was no generation of new bands and the disappearance of existing bands in the SDS-PAGE pattern, indicating that the addition of organic acids induced no formation of new protein subunits. However, an increase in 7 S subunit content and a decrease in 11S content in mTG-induced cross-linked SPI hydrogels were found after treating with GDL, LBA, or MBA. It seemed that the treatment with these organic acids might disrupt the covalent or non-covalent interactions between SPI molecules, resulting in the dissociation of the SPI subunits into fragments with lower molecular weights [57].

### 3.6. Effects of GDL, LBA, or MBA on the Composition of Intramolecular and Intermolecular Forces in mTG-Induced Cross-Linked SPI Hydrogels

Different intramolecular and intermolecular forces played important roles in the formation of SPI hydrogels, including hydrogen bonding, disulfide bonding, hydrophobic interactions, and their interactions. The effects of GDL, LBA, or MBA on the composition of intramolecular and intermolecular forces in mTG-induced cross-linked SPI hydrogels are presented in Table 2. It was shown that the addition of GDL significantly weakened the hydrogen bonding and disulfide bond interactions as well as strengthened the hydrophobic interactions and hydrogen bonding plus disulfide bond plus hydrophobic interactions in hydrogels. By contrast, treating with LBA or MBA greatly weakened the hydrogen bonding and hydrogen bonding plus disulfide bond plus hydrophobic interactions, as well as strengthened the disulfide bond interactions, hydrogen bonding plus disulfide bond interactions, and hydrogen bonding plus hydrophobic interactions. Notably, hydrophobic interactions, as well as hydrogen bonding plus disulfide bonds plus hydrophobic interactions, were essential in the formation of SPI hydrogels treated with mTG and GDL, while hydrogen bonding plus disulfide bond interactions greatly contributed to the gel network structures of mTG plus LBA or mTG plus MBA treatment. Our results were similar to the findings of Wang et al. [58]; they found hydrogen bonding interacted with disulfide bonding as well as the main forces promoting the formation of hydrogels treated by organic acid. In terms of GDL treatment, hydrophobic interactions played a more important role in the formation of SPI hydrogels than other treatments, which might account for the stronger gel network of the GDL-treated group [59].

### 3.7. Effects of GDL, LBA, or MBA on the Moisture Distribution and Morphological Characteristics of mTG-Induced Cross-Linked SPI Hydrogels

**Figure 5:**
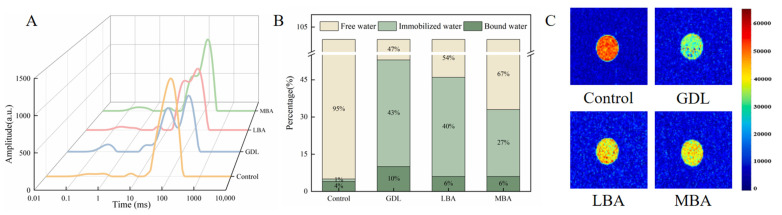
Effects of GDL, LBA, and MBA on the T_2_ curves (**A**), hydrogen proton peak area ratios (**B**), and MRI images (**C**) of mTG-induced cross-linked SPI hydrogels.

The transverse relaxation time (T_2_) of LF-NMR analysis can provide key information about the binding state and mobility of water molecules in composite hydrogel systems [60]. Furthermore, the T_2_ value of samples indirectly indicates the interaction strength between water and solute molecules, which is helpful for assessing the integrity of gel networks. Generally, T_2_ represents the spin relaxation time of samples. Figure 5A showed that four different peaks of T_2_ distribution could be observed for all groups, including the peaks related to strongly bound water (T_2b_), weakly bound water (T_21_), immobilized water (T_22_), and free water (T_23_). In particular, treating with GDL, LBA, or MBA significantly reduced the intensity of the T_22_ peak in mTG-induced cross-linked SPI hydrogels. It could be inferred that the addition of three organic acids made the water molecules in samples become less mobile and tightly bound, leading to the enhanced WHC of hydrogels [61]. The results were in accordance with those in our previous WHC analysis of samples. As shown in Figure 5B, the addition of three organic acids remarkably increased the ratios of immobilized water and bound water in samples. It was worthwhile to note that the immobilized water or bound water content in mTG plus GDL treatment was 43- or 2.5-fold that in the control, respectively. Hence, GDL, LBA, and MBA exhibited promoting effects of converting the free water into immobilized and bound water in mTG-induced cross-linked SPI hydrogels, further improving their water-holding performance [62].

MRI is a non-destructive detecting technique to reflect the difference in moisture distribution in samples. In MRI images, the levels of immobilized water and free water in hydrogels can be seen [63]. As shown in Figure 5C, the color bar changed from red to green and then to blue, suggesting that the hydrogen proton density (free water content) of samples gradually decreased [64]. Similar to the study of Cui et al. [65], the red area of SPI hydrogels significantly decreased after adding three organic acids, while the yellow and green areas became larger. The green area of mTG plus GDL treatment was the largest among all groups. This distinct color gradient shift in mTG plus GDL hydrogels implies a more efficient transition from free to immobilized water phases compared with LBA or MBA treatments, likely driven by their synergistic cross-linking mechanisms. In line with the results of LF-NMR analysis, GDL, LBA, and MBA treatment significantly reduced the water mobility of hydrogels, accounting for their high WHC.

**Figure 6:**
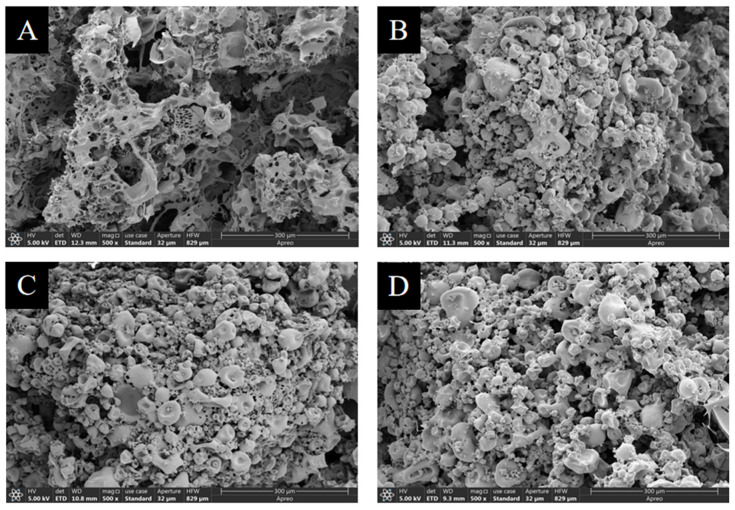
The morphological characteristics of control (**A**) and mTG-induced cross-linked SPI hydrogels treated by GDL (**B**), LBA (**C**), or MBA (**D**).

SEM analysis was utilized to evaluate the effects of three organic acids on the microstructures of mTG-induced cross-linked SPI hydrogels. There were significant differences in the morphologies between the control and the hydrogels treated by GDL, LBA, or MBA (Figure 6). It was observed that the hydrogel particles in the control were loose with large and irregular pores, while the addition of three organic acids reduced the pore size of hydrogel particles, bridged different particles, and made them become more compact, which exhibited a dense stacked aggregate structure. Our findings were similar to the results of Huang et al. [66] and Li et al. [67]; the phenomenon might be attributed to the covalent and non-covalent interactions of organic acids with SPI to fill the pores of SPI hydrogels and further result in a denser microstructure. Consequently, the addition of three organic acids was able to promote the formation of a denser structure of mTG-induced cross-linked SPI hydrogels.

## 4. Conclusions

In the present study, the promoting effects of different organic acids (GDL, CA, MA, SA, LBA, and MBA) on the formation of mTG-induced cross-linked SPI hydrogels, as well as the involving mechanism, were investigated. Our results showed that the gel strength, textural properties, and WHC of samples could be remarkably improved by treating with GDL, LBA, and MBA, while the optimum concentration of GDL, LBA, or MBA to fabricate hydrogels was 1.0% (*w*/*v*), 0.8% (*w*/*v*), or 1.0% (*w*/*v*), respectively. The treatment with organic acids remarkably reduced the H_0_ and intrinsic fluorescence as well as increased the G′ and G″ values, particle size, and the absolute value of negative zeta potential of SPI hydrogels due to their enhancing effects on protein–protein intermolecular hydrophobic interactions and reducing effects on the positive charge on the surface of SPI. Based on the data of FT-IR, SDS-PAGE, and intramolecular and intermolecular forces analysis, the covalent interactions, hydrogen bonding, and hydrophobic interactions of three organic acids with SPI, as well as the effects of organic acids on the interactions among the intramolecular and intermolecular forces of SPI molecules, might play crucial roles in their promoting effects on the formation of hydrogels with high gel strength and stability, while the incorporation of GDL, LBA, or MBA could significantly increase the β-sheet content and decrease the α-helix content in hydrogels, as well as dissociate 11S subunits of SPI into 7S subunits. In terms of the effects of three organic acids on the moisture distribution of samples, GDL, LBA, and MBA were able to reduce the water mobility of hydrogels and help convert the free water into immobilized and bound water to improve their WHC. The SEM analysis confirmed the effects of the addition of three organic acids on reducing the pore size of hydrogel particles and bridging them to form a dense stacked aggregate structure. It was worth mentioning that GDL showed a stronger ability to improve the gelling properties and stability of hydrogels than LBA and MBA, demonstrating superior performance through a significantly higher storage modulus, more favorable water state distribution, and optimal secondary structure composition. Consequently, GDL, LBA, and MBA are promising agents for enhancing the gel strength and stability of mTG-induced cross-linked SPI hydrogels, which was meaningful for broadening the applications of SPI hydrogels in the food industry in the future. It should be noted that while this study shows promising results, some limitations need to be addressed. The long-term stability of these hydrogels under various storage conditions, particularly regarding temperature and humidity fluctuations, requires further investigation. Additionally, the cost-effectiveness and scalability of production processes need systematic evaluation to facilitate potential commercialization.

## Figures and Tables

**Table 1:** Effect of different organic acids on the textural properties of mTG-induced cross-linked SPI hydrogels.

Samples	Hardness (N)	Cohesiveness (%)	Springiness (mm)	Gumminess (N)
Control	4.17 ± 0.13	0.17 ± 0.05	2.95 ± 0.22	1.27 ± 0.00
GDL treatment				
GDL0.2%	3.90 ± 0.24 ^b^	0.27 ± 0.00 ^bc^	8.97 ± 0.08 ^b^	1.03 ± 0.06 ^d^
GDL0.4%	7.62 ± 0.57 ^a^	0.30 ± 0.06 ^ab^	10.48 ± 1.01 ^ab^	2.80 ± 0.10 ^c^
GDL0.6%	12.85 ± 3.28 ^a^	0.24 ± 0.07 ^bc^	10.59 ± 1.02 ^ab^	3.24 ± 0.24 ^c^
GDL0.8%	14.42 ± 1.74 ^a^	0.32 ± 0.00 ^ab^	12.06 ± 1.26 ^a^	4.68 ± 0.75^b^
GDL1.0%	15.66 ± 0.70 ^a^	0.42 ± 0.07 ^a^	12.58 ± 0.43 ^b^	7.89 ± 0.96 ^a^
CA treatment				
CA0.2%	3.24 ± 0.17 ^b^	0.25 ± 0.03 ^abc^	4.15 ± 0.09 ^a^	0.79 ± 0.05 ^bc^
CA 0.4%	3.01 ± 0.13 ^bc^	0.22 ± 0.01 ^bc^	3.73 ± 0.47 ^ab^	0.66 ± 0.01 ^c^
CA 0.6%	2.52 ± 0.40 ^c^	0.28 ± 0.01 ^ab^	4.58 ± 0.66 ^a^	0.81 ± 0.08 ^bc^
CA 0.8%	2.86 ± 0.18 ^bc^	0.35 ± 0.06 ^a^	4.34 ± 0.27 ^a^	0.99 ± 0.09 ^b^
CA 1.0%	2.65 ± 0.04 ^c^	0.26 ± 0.04 ^ab^	3.98 ± 0.21 ^a^	0.84 ± 0.15 ^bc^
MA treatment				
MA0.2%	4.25 ± 0.23 ^a^	0.25 ± 0.02 ^ab^	7.97 ± 0.71 ^a^	1.10 ± 0.06 ^a^
MA0.4%	3.27 ± 0.20 ^b^	0.21 ± 0.02 ^ab^	3.98 ± 0.51 ^bc^	0.82 ± 0.01 ^bc^
MA0.6%	3.21 ± 0.27 ^b^	0.28 ± 0.04 ^a^	3.96 ± 1.12 ^bc^	0.90 ± 0.14 ^b^
MA0.8%	1.72 ± 0.16 ^c^	0.24 ± 0.05 ^ab^	4.56 ± 0.90 ^bc^	0.50 ± 0.07 ^d^
MA1.0%	1.70 ± 0.02 ^c^	0.26 ± 0.01 ^ab^	6.01 ± 1.14 ^ab^	0.67 ± 0.09 ^cd^
SA treatment				
SA0.2%	4.14 ± 0.05 ^a^	0.32 ± 0.03 ^a^	9.62 ± 0.47 ^a^	1.20 ± 0.07 ^a^
SA0.4%	2.02 ± 1.11 ^b^	0.30 ± 0.01 ^a^	3.76 ± 0.45 ^bc^	0.82 ± 0.07 ^b^
SA0.6%	1.27 ± 0.69 ^b^	0.28 ± 0.06 ^a^	3.04 ± 0.31 ^c^	0.61 ± 0.06 ^c^
SA0.8%	1.74 ± 0.67 ^b^	0.31 ± 0.03 ^a^	4.70 ± 0.99 ^b^	0.54 ± 0.02 ^c^
SA1.0%	1.25 ± 0.07 ^b^	0.37 ± 0.07 ^a^	2.76 ± 0.38 ^c^	0.40 ± 0.03 ^d^
LBA treatment				
LBA0.2%	3.68 ± 0.07 ^c^	0.31 ± 0.12 ^ab^	6.57 ± 0.15 ^c^	1.02 ± 0.15 ^d^
LBA 0.4%	3.81 ± 0.15 ^c^	0.30 ± 0.04 ^b^	8.17 ± 0.41 ^b^	1.04 ± 0.23 ^d^
LBA 0.6%	5.81 ± 0.71 ^b^	0.33 ± 0.04 ^ab^	9.50 ± 0.99 ^ab^	1.92 ± 0.02 ^c^
LBA 0.8%	10.12 ± 0.95 ^a^	0.52 ± 0.16 ^a^	10.12 ± 0.16 ^a^	4.70 ± 0.43 ^a^
LBA 1.0%	9.25 ± 0.18 ^a^	0.27 ± 0.02 ^b^	10.57 ± 0.73 ^a^	2.98 ± 0.45 ^b^
MBA treatment				
MBA0.2%	3.44 ± 0.37 ^c^	0.35 ± 0.03 ^bc^	9.51 ± 0.72 ^c^	1.21 ± 0.03 ^bc^
MBA 0.4%	3.77 ± 0.37 ^bc^	0.30 ± 0.02 ^c^	10.20 ± 0.56 ^bc^	1.14 ± 0.05 ^c^
MBA 0.6%	4.41 ± 0.63 ^bc^	0.40 ± 0.04 ^ab^	10.43 ± 0.14 ^bc^	1.50 ± 0.06 ^b^
MBA 0.8%	4.99 ± 0.87 ^ab^	0.47 ± 0.03 ^a^	10.95 ± 0.23 ^b^	2.34 ± 0.24 ^a^
MBA 1.0%	5.97 ± 0.80 ^a^	0.32 ± 0.03 ^bc^	14.73 ± 0.26 ^a^	2.13 ± 0.14 ^a^

Data are expressed as mean ± SD (*n* = 3); the statistical significance of data of each group and control was determined using one-way ANOVA followed by the comparison of DMRT, while data marked with the same letter showed no significant difference at *p* < 0.05.

**Table 2:** Effect of GDL, LBA, and MBA on the composition of intramolecular and intermolecular forces in mTG-induced cross-linked SPI hydrogels.

Chemicalbonds	Hydrogen Bonds	Disulfide Bonds	Hydrophobic Interactions	Hydrogen Bonds + Disulfide Bonds	Hydrogen Bonds + Hydrophobic Interactions	Hydrophobic Interactions + Disulfide Bonds	Hydrogen Bonds + Disulfide Bonds + Hydrophobic Interactions
Control	2.22 ± 0.50 ^a^	33.77 ± 1.22 ^b^	1.64 ± 0.35 ^b^	0.31 ± 0.02 ^c^	−1.10 ± 0.54 ^b^	−12.73 ± 4.33 ^bc^	32.52 ± 5.49 ^b^
GDL	0.97 ± 0.06 ^b^	22.22 ± 2.27 ^c^	7.67 ± 1.42 ^a^	7.00 ± 1.88 ^c^	−0.81 ± 0.15 ^b^	−7.27 ± 2.07 ^b^	57.10 ± 11.16 ^a^
LBA	0.71 ± 0.27 ^bc^	41.94 ± 5.64 ^a^	0.81 ± 0.46 ^b^	58.15 ± 6.05 ^b^	1.69 ± 0.13 ^a^	−26.45 ± 5.38 ^c^	−39.32 ± 5.59 ^c^
MBA	0.25 ± 0.18 ^c^	46.79 ± 5.07 ^a^	1.94 ± 0.66 ^b^	98.05 ± 5.28 ^a^	2.17 ± 0.40 ^a^	69.59 ± 13.66 ^a^	−140.51 ± 9.56 ^d^

Data are expressed as mean ± SD (*n* = 3); the statistical significance of data of each group and control was determined using one-way ANOVA followed by the comparison of DMRT, while data marked with the same letter showed no significant difference at *p* < 0.05.

## Data Availability

The original contributions presented in this study are included in the article. Further inquiries can be directed to the corresponding author.
